# Real-world experience with pembrolizumab toxicities in advanced melanoma patients: a single-center experience in the UK

**DOI:** 10.2217/mmt-2017-0028

**Published:** 2018-04-24

**Authors:** Alfred CP So, Ruth E Board

**Affiliations:** 1 Faculty of Biology, Medicine, & Health, The University of Manchester, Oxford Road, Manchester, M13 9PL, UK; 2 Oncology Department, Lancashire Teaching Hospitals NHS Trust, Sharoe Green Lane, Fulwood, Preston, PR2 9HT, UK

**Keywords:** anti-PD-1 therapy, immune-related adverse events, melanoma, pembrolizumab, real-world experience, retrospective study

## Abstract

**Aim:**

We aimed to characterize the safety profile of pembrolizumab in advanced melanoma patients at our center to better reflect ‘real-world’ data on anti-PD-1 inhibitors.

**Materials & methods:**

At our institution, 58 ipilimumab-naive and 30 ipilimumab-treated patients with advanced melanoma who have received pembrolizumab between June 2014 and June 2017 were included for analysis.

**Results:**

Incidence of any-grade and grade 3/4 toxicities were 81.8% (n = 72) and 12.5% (n = 11), respectively. The most common side effects were skin-related (61.4%, n = 54) and gastrointestinal-related (51.1%, n = 45) events. In total, 25% of patients required oral steroids to manage immune-related adverse events with a median cumulative prednisolone dose of 683 mg (range: 40–3745 mg).

**Conclusion:**

Pembrolizumab is well tolerated in ‘real-world’ patients and severe toxicities can be effectively managed with systemic steroids.

**Practice points**

- Pembrolizumab is generally well tolerated by advanced melanoma patients.
- Skin-related and gastrointestinal-related events are the most common immune-related adverse events (irAEs) experienced with pembrolizumab.
- Endocrine toxicities, such as hypothyroidism, are a permanent side effect of pembrolizumab, and patients will require lifelong replacement with thyroxine.
- Many patients will require systemic steroids at some point during their treatment to manage immune-irAEs, the long-term implications of which are still uncertain.
- We observed higher incidences of irAEs in our patients compared with pivotal trials, reflecting the underrepresentation of patients in clinical studies.
- More ‘real-world’ data from observational studies is required to guide clinical decision-making and information provision to patients.
- Further work is needed to clarify the long-term effects of anti-PD-1 therapy.

Melanoma is the fifth most common cancer and the 18th most common cause of cancer death in the UK [1]. Before the advent of checkpoint inhibitors, treatment options for metastatic melanoma were limited and patients had poor survival rates with median overall survival (OS) of ≤1 year [2]. The use of checkpoint inhibitors has extended both progression-free survival and OS in advanced melanoma patients (stage III/IV) [3,4]. Furthermore, the approvals of anti-PD-1 antibodies such as pembrolizumab and nivolumab have since superseded ipilimumab (an anti-CTLA-4 antibody) as the current standard first-line treatment in the UK for many patients with metastatic melanoma due to their superior efficacies and favorable toxicity profiles.

The regulation of T-cell activation through checkpoint pathways is an important part of self-tolerance. This discrimination between self and nonself is essential to prevent immune-mediated tissue damage. However, as checkpoint inhibitors block these pathways, overstimulation of the immune system can overcome self-tolerance resulting in inflammatory responses termed immune-related adverse events (irAEs). The most common nonimmune adverse events (AEs) of checkpoint inhibitors are fatigue, diarrhea, pruritus, and nausea [3–7]. However, irAEs can occur in any tissues most commonly the skin, GI tract, thyroid gland, pituitary gland, lungs and liver [8].

Although the range of irAEs is quite similar across checkpoint inhibitors, there are differences in frequency of toxicities. Grade 3/4 toxicities are less commonly observed with PD-1 inhibitors compared with CTLA-4 inhibitors [8]. Furthermore, PD-1 inhibitors have higher rates of thyroiditis and pneumonitis compared with CTLA-4 inhibitors [8]. Other factors that may influence toxicity profiles include combination therapies, treatment sequencing, tumor type and preexisting autoimmune conditions such as rheumatoid arthritis and inflammatory bowel disease [9,10].

Pembrolizumab is generally well tolerated but severe irAEs can lead to significant patient morbidity. Majority of grade 3/4 irAEs can be managed with oral (and intravenous) steroids. In steroid-refractory cases, other immunomodulatory drugs may be used including infliximab, mycophenolate mofetil and calcineurin inhibitors [11].

It is a common concern among clinicians that the incidence of AEs in clinical trials do not necessarily reflect ‘real-world’ experiences due to underrepresentation of the patient population [12]. While early identification of AEs is dependent on both the clinician and self-reporting by patients, underrepresented data can hinder effective clinical practice. Moreover, published real-life data on irAEs with pembrolizumab are limited. In the UK, the PD-1 inhibitors, pembrolizumab and nivolumab, are funded by the NHS for use in melanoma. The majority of patients in our institution receive pembrolizumab rather than nivolumab due to its less intense 3-weekly schedule (2-weekly for nivolumab). In this study, we analyzed the incidence and management of pembrolizumab-associated irAEs in our hospital to guide medical decision-making and health information provision for patients.

## Materials & methods

### Patient sample

We retrospectively analyzed the data on all patients with advanced melanoma who received pembrolizumab (2 mg/kg every 3 weeks) at a single UK center between 15th June 2014 and 15th June 2017. Inclusion criteria included adequate documentation of the treatment course and histologically confirmed advanced cutaneous melanoma. Patients who received treatment as part of an expanded patient access program were also included (n = 5). Patient-specific information was collected using our hospital's electronic databases. Data collected included patient demographics, tumor characteristics (Tumor, Node, Metastasis (TNM) staging, brain metastasis, baseline LDH, *BRAF* ^*V600*^ status), previous systemic therapies, date of first immunotherapy dose, date of death (or the last follow-up), baseline WHO performance status (PS), number of treatment doses and deferrals, best overall response and AEs. Data were also collected on the management of irAEs including cumulative steroid dose.

### Data analysis

AEs were graded using the Common Terminology Criteria for Adverse Events v4.0. Select AEs of interest were categorized into organ-specific groups for analysis. Of note, ‘general’ AEs encompass nonspecific symptoms including fatigue/lethargy, asthenia, pyrexia, and decreased appetite. Due to the limitations of retrospective grading of toxicities, diarrhea and colitis were grouped together. AEs were grouped into grades 1/2 and 3/4 due to inherent difficulties in retrospective grading from some clinical notes.

Estimated total steroid dose per patient was calculated based on documented prescriptions and steroid-tapering guidelines. All steroids were converted to the equivalent prednisolone dose for ease of comparison. Any steroids prescribed for hormone-replacement therapy in adrenal insufficiency and for non-irAEs, such as brain metastasis and radiation-induced pneumonitis, were excluded from analysis. Time to first grade 3/4 AE was defined as time from first treatment dose to time of first reported AE. Patients who did not experience grade 3/4 AEs were censored at time of last follow-up. OS was defined as time from first dose of pembrolizumab to death of any cause. Patients who were still alive at data cutoff were censored at time of the last follow-up. Median follow-up time was defined as time from first treatment dose to time of the last follow-up. Patients who have died prior to data cutoff were censored at their date of death. Best overall response was identified based on documented responses, and was not formally assessed retrospectively using the immune-related Response Evaluation Criteria In Solid Tumors (ir-RECIST). The TNM staging system used was version 7 of the American Joint Committee on Cancer staging (AJCC). Kaplan–Meier survival curves were produced using GraphPad Prism 7. 95% CI were calculated using the Anderson's method (1993). We compared the incidence of AEs in the study groups using the Chi-square test, where p = 0.05 was considered statistically significant.

## Results

### Patient demographics

A total of 88 patients with melanoma were identified as suitable for inclusion and analysis. In total, 58 patients received pembrolizumab as first-line immunotherapy (ipilimumab-naive group) and 30 patients received pembrolizumab following ipilimumab therapy (ipilimumab-treated group). Demographics are shown in Table 1. The median age was 69 years (range: 21–91) and 61.5 years (range: 31–86) old in ipilimumab-naive and ipilimumab-treated patients, respectively. Of the total 88 patients, 62.5% were male, 92.0% had a PS of 0–1, 61.4% had stage M1c disease and 12.5% had brain metastases. 40.9% of the patients were positive for *BRAF* ^*V600*^ mutation, of which 36.1% had received prior BRAF ± MEK inhibitor therapy. 89.7% of ipilimumab-naive patients have not received prior systemic treatments for their melanoma. Baseline LDH levels were unknown in 46 patients, the majority due to hemolysed blood samples. We recognize that this can affect staging, however, 18 out of 46 patients’ staging would not have been affected by LDH levels as they were already staged M1c disease. At data cutoff, the median duration of follow-up in the ipilimumab-naive and ipilimumab-treated cohorts were 12 months (range: 0.8–18.3) and 23 months (range: 4.5–31.3), respectively.

**Table 1.** **Patient demographics.**

**Characteristics**	**Subcategory**	**Ipilimumab-naive (n = 58), No. (%)**	**Ipilimumab-treated (n = 30), No. (%)**
Age (years)	Median	69	61.5

	Range	21–91	31–86

Sex	Male	38 (65.5)	17 (56.7)

	Female	20 (34.5)	13 (43.3)

ECOG PS	0	39 (67.2)	17 (56.7)

	1	14 (24.1)	11 (36.7)

	≥2	5 (8.6)	1 (3.3)

	Not reported	0	1 (3.3)

Metastasis	M0, M1a, M1b	25 (43.1)	9 (30.0)

	M1c	33 (56.9)	21 (70.0)

Brain metastases	Yes	5 (8.6)	6 (20.0)

	No	53 (91.4)	23 (80.0)

LDH	Normal	8 (13.8)	7 (23.3)

	Raised	15 (25.9)	12 (40.0)

	Unknown	35 (60.3)	11 (36.7)

*BRAF*^*V600*^	Mutation	25 (43.1)	11 (36.7)

	Wild-type	33 (56.9)	19 (63.3)

Number of previous systemic treatment(s)	0	52 (89.7)	0

	1	6 (10.3)	20 (66.7)

	≥2	0	10 (33.3)

Previous treatment	Ipilimumab	0	30 (100.0)

	Chemotherapy	0	3 (10.0)

	BRAF ± MEK	6 (10.3)	7 (23.3)

ECOG PS: Eastern cooperative oncology group performance status.

### Adverse events

The incidence of any-grade and grade 3/4 AEs were 77.6 and 10.3% in ipilimumab-naive patients, and 90.0 and 16.7% in ipilimumab-treated patients (Table 2). No treatment-related deaths were observed. The most common any-grade AEs in ipilimumab-naive and ipilimumab-treated patients were fatigue/lethargy (34.5 and 26.7%), pruritus (20.7 and 36.4%), rash (22.4 and 40.0%), diarrhea/colitis (26.8 and 46.7%), endocrine-related toxicities (29.3 and 26.7%) and musculoskeletal-related toxicities (19.0 and 20.0%). Compared with ipilimumab-naive patients, ipilimumab-treated patients experienced significantly higher incidences of any-grade skin toxicities (90.0 vs 46.6%; p < 0.01) and diarrhea/colitis (46.7 vs 26.8%; p = 0.028). Grade 3/4 diarrhea/colitis was observed in 3.4 and 13.3% of ipilimumab-naive and ipilimumab-treated patients, respectively. Of interest, three patients were diagnosed with biopsy proven immunotherapy-related sarcoidosis, of which one patient was symptomatic from a skin lesion and required oral steroids. All three patients were incidentally diagnosed on follow-up CT to assess for treatment response and were shown to have new bilateral hilar lymphadenopathy and mediastinal lymphadenopathy. All patients had raised ACE levels and endobronchial ultrasound-guided biopsy was performed on all patients to rule out new metastatic lesions and confirmed the histological diagnosis of sarcoidosis.

**Table 2.** **Select adverse events of interest.**

**AEs**	**Ipilimumab-naive (n = 58), No. (%)**		**Ipilimumab-treated (n = 30), No. (%)**		**p-value**			

	**Any grade**	**Grade 3/4**	**Any grade**	**Grade 3/4**	**Any grade**	**Grade 3/4**		
Any AEs	45 (77.6)	6 (10.3)	27 (90.0)	5 (16.7)	0.071	0.150

General	25 (43.1)	1 (1.7)	11 (36.7)	0	0.187	0.168

Skin	27 (46.6)	1 (1.7)	27 (90.0)	0	<0.001	0.168

– Pruritus	12 (20.7)	0	11 (36.7)	0	0.053	–

– Rash	13 (22.4)	1 (1.7)	12 (40.0)	0	0.045	0.168

Gastrointestinal	28 (48.3)	2 (3.4)	17 (56.7)	4 (13.3)	0.165	0.042

– Diarrhea/colitis	15 (26.8)	2 (3.4)	14 (46.7)	4 (13.3)	0.028	0.042

HPB	6 (10.3)	0	4 (13.3)	1 (3.3)	0.206	0.075

– Hepatitis	5 (8.6)	0	4 (13.3)	1 (3.3)	0.173	0.075

– Pancreatitis	1 (1.7)	0	0	0	0.168	–

Endocrine	17 (29.3)	0	8 (26.7)	0	0.221	–

– Hypothyroidism	11 (19.0)	0	4 (13.3)	0	0.176	–

– Hyperthyroidism	6 (10.3)	0	2 (6.7)	0	0.189	–

– Hypoadrenalism	0	0	2 (6.7)	0	0.027	–

Pulmonary	4 (6.9)	0	1 (3.3)	0	0.174	–

– Pneumonitis	4 (6.9)	0	1 (3.3)	0	0.174	–

Renal	1 (1.7)	0	2 (6.7)	0	0.098	–

Neurological	2 (3.4)	0	1 (3.3)	0	0.230	–

Musculoskeletal	11 (19.0)	2 (3.4)	6 (20.0)	0	0.149	0.124

– Arthritis	3 (5.2)	1 (1.7)	3 (10.0)	0	0.149	0.168

– Myositis	1 (1.7)	1 (1.7)	0	0	0.168	0.168

Eye	0	0	2 (6.7)	0	0.027	–

Hematological	5 (8.6)	0	4 (13.3)	0	0.173	–

Others	1 (1.7)	0	2 (6.7)	0	0.098	–

– Sarcoidosis	1 (1.7)	0	2 (6.7)	0	0.098	–

p-values were calculated using Chi-square test.

AE: Adverse event; HPB: Hepatopancreato-biliary.

Of the total 88 patients, 15 patients (17.0%) were diagnosed with hypothyroidism during treatment, of which, eight patients (53.3%) were preceded with biochemical hyperthyroidism. Only one of these eight patients was symptomatic, requiring treatment with β-blockers. Furthermore, two ipilimumab-treated patients required long-term steroid replacement due to primary and secondary adrenal insufficiency.

### Time to adverse event

**Figure 1.**
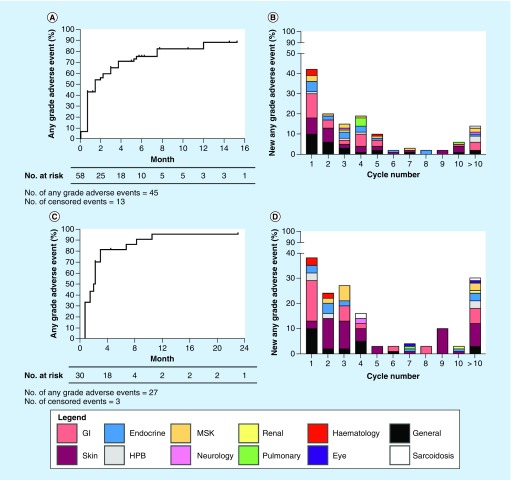
**Any-grade adverse events.** Time to first any-grade immune-related adverse event (irAE) in **(A)** ipilimumab-naive and **(C)** ipilimumab-treated patients. New any-grade irAE in relation to treatment cycle number in **(B)** ipilimumab-naive and **(D)** ipilimumab-treated patients. The median durations to any-grade adverse event were 1.5 and 2.0 months in ipilimumab-naive **(A)** and ipilimumab-treated patients **(C)**, respectively. GI: Gastrointestinal; HPB: Hepatopancreato-biliary; MSK: Musculoskeletal.

Median times to any grade AE were 1.5 months (95% CI: 0.7–2.3) and 2.0 months (95% CI: 1.5–2.5) in ipilimumab-naive and ipilimumab-treated patients, respectively (Figure 1A & C). The majority of grade 3/4 AEs occurred within 4 months of starting pembrolizumab. 43.1 and 36.7% of ipilimumab-naive and ipilimumab-treated patients, respectively, experienced their first any-grade AE after one cycle of treatment. Gastrointestinal-related toxicities were the most common AE postcycle 1 in ipilimumab-naive (28.9%) and ipilimumab-treated (42.9%) patients. The majority of new AEs occurred between cycles 1 and 4 in all cohorts with the frequency of new events gradually decreasing as treatment time progressed. In the ipilimumab-treated group, there was a rise in the number of new skin-related toxicities from cycle 2 onward. It should be noted that a number of side effects did not occur until after ten cycles of treatment.

### Management of irAEs

Of the 88 patients, 22 patients required oral steroids to manage side effects of pembrolizumab (ipilimumab-naive, n = 12 [20.7%] and ipilimumab-treated, n = 10 [33.3%]). Although systemic steroids are primarily used in the management of grade 3/4 irAEs, we observed a number of patients with grade 1/2 irAEs requiring systemic steroids for persistent symptoms. In our series, no patients required further management with infliximab or other immunosuppressive agents. However, one patient required admission for intravenous steroids to treat grade 3/4 colitis.

All patients on systemic treatment for irAEs experienced symptomatic relief. The median total duration and cumulative dose of systemic steroids used to manage irAEs were 31.5 days (range: 1–259 days) and 683 mg (range: 40–3745 mg), respectively. One patient developed diabetes due to prolonged exposure to high-dose steroids (estimated cumulative dose of prednisolone = 630 mg; Table 3). There were two patients who were unable to wean off steroids at data cutoff due to ongoing symptoms from immune-related arthritis.

**Table 3.** **Management of immune-related adverse events.**

**Variable**	**Ipilimumab-naive (n = 58), No. (%)**	**Ipilimumab-treated (n = 30), No. (%)**
Patients requiring immunosuppresion	12 (20.7)	10 (33.3)

Type of immunosuppression		

– Steroid (oral)	12 (20.7)	10 (33.3)

– Steroid (IV)	1 (1.7)	0

Estimated total duration of steroid treatment		

– Mean (SD)	49.2 (40.3)	60.3 (73.5)

Median (range)	40 (1–122)	29.5 (3–259)

Estimated cumulative prednisolone dose		

– Mean (SD)	1057.7 (1074.6)	1206 (1202.8)

– Median (range)	587.5 (120–2257.5)	965 (40–3745)

IV: Intravenous; SD: Standard deviation.

### Patients with autoimmune conditions

Fourteen patients had autoimmune conditions prior to the start of their treatment, which included polymyalgia (n = 1), rheumatoid arthritis (n = 2), seronegative-inflammatory arthritis (n = 1), psoriasis (n = 1), Crohn's disease (n = 1), ulcerative colitis (n = 1), thyroid peroxidase antibody (TPO)-positive thyroiditis (n = 1), lichen sclerosus (n = 1) and sarcoidosis (n = 1). 11 of the 14 (78.6%) patients had flare-ups of their underlying conditions on pembrolizumab. These were: polymyalgia, rheumatoid arthritis, seronegative-inflammatory arthritis, Crohn's disease, ulcerative colitis, and TPO-positive thyroiditis. Oral steroids were used to manage these ‘flare-ups’ of autoimmune disease with good effect, particularly with respect to arthritis. The patient with preexisting ulcerative colitis required intravenous steroids to treat grade 3/4 colitis unresponsive to oral steroids.

### Treatment response

The median numbers of treatment cycles administered to ipilimumab-naive and ipilimumab-treated patients were six (range: 2–26) and 10 (range: 2–38), respectively; the proportions of patients continuing treatment at the time of data cutoff were 58.6 and 20.0%, respectively. Three patients permanently discontinued their treatment due to immune-related skin rash (n = 1), pneumonitis (n = 1) and sarcoidosis (n = 1). The proportions of ipilimumab-naive and ipilimumab-treated patients requiring treatment deferral were 29.3 and 43.3%, respectively, of which 36.4 and 35.0% were related to treatment toxicity, respectively. Patient choice, such as vacation plans, was also a major contributing factor to treatment deferral.

**Figure 2.**
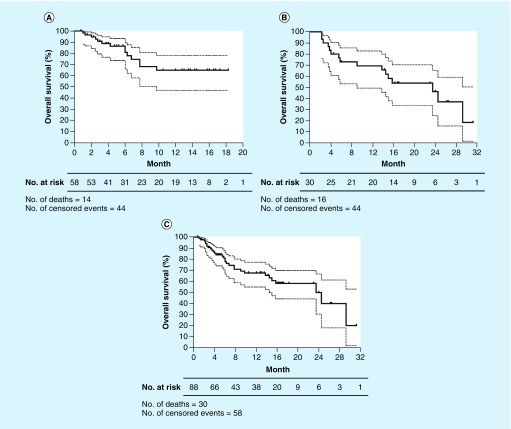
**Overall survival rates.** Kaplam–Meier estimates of **(A)** ipilimumab-naive patients and **(B)** ipilimumab-treated patients, and **(C)** all patients. The median overall survival was 23.5 months in the ipilimumab-treated patients **(B)** and not reached in the ipilimumab-naive **(A)** patients. Dashed lines represent 95% CI.

A total of 30 deaths occurred at the time of data cutoff. Overall response rates (ORRs) in ipilimumab-naive and ipilimumab-treated patients were 48.3% (95% CI: 40.1–56.5) and 50.0% (95% CI: 37.3–62.7), respectively. Median OS was 23.5 months (95% CI: 13.9–33.1) in the ipilimumab-treated cohort, and was not reached in the ipilimumab-naive cohort. One-year estimates of survival were 64.9% (95% CI: 47.1–78.1) and 69.4% (95% CI: 49.3–82.8) in ipilimumab-naive and ipilimumab-treated patients, respectively. The median OS and 1-year survival rate in all patients were 23.5 months (95% CI: 22.0–25.0) and 67.5% (95% CI: 54.9–77.3), respectively (Figure 2 and Table 4). Further statistical analyses were not performed on the survival data as the differences in ORR is minimal and unlikely to be of clinical significance.

**Table 4.** **Treatment response.**

**Variable**	**Subcategory**	**Ipilimumab-naive (n = 58), No. (%)**	**Ipilimumab-treated (n = 30), No. (%)**
BOR – number (%)	Complete response	1 (1.7)	0

	Partial response	27 (46.6)	15 (50.0)

	Stable disease	7 (12.1)	5 (16.7)

	Progressive disease	20 (34.5)	9 (30.0)

	Not determined	3 (5.2)	1 (3.3)

ORR (complete + partial response)	Number	28	15

	% (95% CI)	48.3 (40.1–56.5)	50.0 (37.3–62.7)

	Median OS in months (95% CI)	Not reached	23.5 (13.9–33.1)

	1-year estimates of survival % (95% CI)	64.9 (47.1–78.1)	69.4 (49.3–82.8)

BOR: Best overall response; ORR: Overall response rate; OS: Overall survival.

Having a preexisting autoimmune condition, receiving systemic steroids for irAEs, or having a *BRAF*-mutant positive status, did not appear to significantly affect response to pembrolizumab. A comparison of median OS in patients who had one of these factors to patients who did not was not possible, as the median OS was not reached in the majority of the subpopulations. Median OS of ipilimumab-treated patients who received systemic steroids for irAEs was lower compared with those who did not receive steroids (5.75 vs 24.0 months, respectively). However, we recommend that these comparisons should be interpreted with caution due to our small sample size.

## Discussion

The success of immunotherapies has led to pembrolizumab being established as a successful treatment option for advanced melanoma. However, there are currently limited published data on ‘real-world’ experiences with pembrolizumab in melanoma patients, particularly the incidence and management of irAEs. The primary objective of this study was to characterize our center's experience of pembrolizumab use. The KEYNOTE-002 and KEYNOTE-006 clinical trials were used as comparators as they demonstrated superiority of pembrolizumab against the previous standard of care in ipilimumab-treated and ipilimumab-naive patients, respectively [3,4].

Patient baseline characteristics in both cohorts were comparable to pivotal studies with regard to age, gender, and PS. There was a higher incidence of brain metastases in our series than the clinical trials, which is to be expected in a real-world population. Interestingly, our cohorts had less M1c disease at baseline and were less heavily pretreated compared with trial participants. ORRs in ipilimumab-naive and ipilimumab-treated patients were 48.3% (95% CI: 40.1–56.5) and 50.0% (95% CI: 37.3–62.7) compared with 32.9% (95% CI: 27.4–38.7) and 21.1% (95% CI: 15–28) in KEYNOTE-006 and KEYNOTE-002, respectively [3,4]. Although ORRs were more favorable in our cohort, this could be a result of not formally assessing treatment response using the (ir)RECIST criteria in clinical practice, thus reflecting one of the limitations with retrospective studies. One-year estimates of survival were similar between our ipilimumab-naive cohort (64.9%; 95% CI: 47–1–78.1) and KEYNOTE-006 (68.4%; 95% CI: 62.5–73.6) [4]. However, median OS was superior in our ipilimumab-treated cohort (23.5 months; 95% CI: 13.9–33.1) compared with KEYNOTE-002 (13.4 months; 95% CI: 11.0–16.4), which could reflect the better prognostic features of the patients in our series [3].

In ipilimumab-naive patients, overall incidence of total any-grade and grade 3/4 AEs was similar between our cohort (77.6 and 10.3%) and KEYNOTE-006 (72.9 and 10.1%) [5]. However, we observed higher rates of diarrhea/colitis (26.8 vs 18.1%), rash (20.7 vs 14.1%), pruritus (22.4 vs 13.4%), constipation (10.3 vs 1.8%), hypothyroidism (19.0 vs 8.7%), and hyperthyroidism (10.3 vs 3.2%) [4].

In ipilimumab-treated patients, incidence of total any-grade and grade 3/4 irAEs was higher in our cohort (90.0 and 16.7%) compared with KEYNOTE-002 (67.4 and 10.7%) [3]. Specific irAEs that were more prevalent in our cohort included diarrhea/colitis (46.7 vs 9.6%), pruritus (36.7 vs 20.8%), rash (40.0 vs 14.6%), hypothyroidism (13.3 vs 5.1%), and dry skin (10.0 vs 5.1%) [3].

In the Phase II randomized CheckMate-064 trial comparing the safety of sequential administration of nivolumab and ipilimumab, higher incidences of grade 3/4 irAEs were observed in the second induction period compared with the first induction period in either sequences [13]. This suggests that patients who were previously primed to a class of checkpoint inhibitor (e.g., anti-CTLA-4) are at an increased risk of more toxicities with a different class (e.g., anti-PD-1). This may explain the higher incidence of toxicities in our ipilimumab-treated patients compared with ipilimumab-naive patients.

The frequency of oral steroids administered for any-grade irAEs were 12 (20.7%) and 10 (33.3%) in ipilimumab-naive and ipilimumab-treated patients. This is a significant proportion of our cohort requiring steroids for irAEs and one patient developed steroid-related diabetes. The cumulative dose of steroids received was significant and the long-term outcomes and implications of long-term steroid use in this group of patients have been poorly studied. In the retrospective setting, it was difficult to measure other steroid-related AEs such as proximal weakness and weight gain. The short-term and long-term implications of steroid use in cancer patients have already been extensively reviewed in other papers [14,15]. Some of the severe sequelae of chronic steroid use include an increase in the risk of opportunistic infections, metabolic syndrome, adrenal suppression, osteoporosis and neuropsychiatric disorders. Moreover, prolonged steroid use could theoretically reduce the efficacy of checkpoint inhibitors, although this is still an area of uncertainty [16]. We recommend that there should be more research in this area as patients survive for longer after immunotherapy treatment.

There is little published literature documenting real life experiences with pembrolizumab. There are some small retrospective series that also highlight the increased incidence of irAEs in clinical practice in comparison to clinical trials [17–19]. A single-center observational study of 39 ipilimumab-treated patients receiving anti-PD-1 therapy reported any-grade and grade 3/4 irAEs of 48.7 and 15.4%, respectively [20]. Although the incidence of severe irAEs was similar to our results, there was a large difference in any-grade irAEs. One important difference between our results and those in clinical studies is the incidence of thyroid disorders, in particular hypothyroidism. Incidence of hypothyroidism reported in the KEYNOTE-002 and KEYNOTE-006 studies was 5.1 and 8.7%, respectively. In comparison, the incidence of hypothyroidism in our ipilimumab-treated and ipilimumab-naive patients was 13.3 and 19.0%, respectively. This has been similarly reported in a small observational study of ipilimumab-treated patients with a reported incidence of approximately 15% [20]. It is important that thyroid function tests are monitored regularly on pembrolizumab treatment and is important to recognize that this is a permanent side effect.

Early recognition of irAEs is key to management and can potentially reverse it, thus avoiding long-term sequelae [21]. Early identification of irAEs (and non-irAEs) is often dependent on self-reporting by patients. Patient self-reporting of systemic anticancer treatment (SACT) toxicities can occur either in clinician/nurse-led consultations or through a nurse-led 24 h helpline. The UK National Chemotherapy Board has proposed two approaches to promote early identification of SACT toxicities: Empowerment and Proactive monitoring [22]. It is recommended that patients starting on SACT receive information about the treatment and provide appropriate consent. Studies such as ours highlight the need for specific preimmunotherapy information to be given to patients, carers and health professionals involved in the patient's care.

There are inherent limitations in a retrospective study that have to be acknowledged. In particular, accuracy of grading and reporting of irAEs in clinical records and documentation of cumulative steroid exposure. Although these limitations can impact the reliability of the data, our series reflects a more realistic ‘real-world’ experience with pembrolizumab treatment. We were also able to confirm that patients with preexisting autoimmune conditions can be safely treated with PD-1 inhibitors but need to be counseled about the significant risk of exacerbating their underlying autoimmune condition [10]. We recognize that our median follow-up time of 12 months is relatively short. However, the majority of the irAEs appear early on in the treatment course, from within a few weeks to up to 3 months, and therefore a 12-month follow-up is sufficient.

## Conclusion

We observed a higher incidence of irAEs with ‘real-world’ patients with metastatic melanoma. The need for more ‘real-world’ data from observational studies is required to guide clinical decision-making and information provision to patients. Furthermore, with the recent positive results for checkpoint inhibitors in the adjuvant setting and the approval for combination ipilimumab/nivolumab in advanced melanoma patients in the UK, there will an increasing number of patients with melanoma-receiving immunotherapy. Immunotherapy treatments are increasingly licensed in other tumor types, such as lung and renal cancer, and vigilance is needed in identifying and treating these side effects. Further work to clarify the long-term effects of immunotherapy toxicity and treatment is required.
